# Tamsulosin-induced severe hypotension during general anesthesia: a case report

**DOI:** 10.1186/1752-1947-4-365

**Published:** 2010-11-17

**Authors:** Dileep Kumar, Fauzia Anis Khan

**Affiliations:** 1 Department of Anesthesia, Aga Khan University PO Box 3500, Stadium Road, Karachi, Pakistan

## Abstract

**Introduction:**

Tamsulosin, a selective α_1_-adrenergic receptor (α_1_-AR) antagonist, is a widely prescribed first-line agent for benign prostatic hypertrophy (BPH). Its interaction with anesthetic agents has not been described.

**Case presentation:**

We report the case of 54-year-old Asian man undergoing elective left thyroid lobectomy. The only medication the patient was taking was tamsulosin 0.4 mg for the past year for BPH. He developed persistent hypotension during the maintenance phase of anesthesia while receiving oxygen, nitrous oxide and 1% isoflurane. The hypotension could have been attributable to a possible interaction between inhalational anesthetic and tamsulosin.

**Conclusion:**

Vigilance for unexpected hypotension is important in surgical patients who are treated with selective α_1_-AR blockers. If hypotension occurs, vasopressors that act directly on adrenergic receptors could be more effective.

## Introduction

Tamsulosin (benzenesulfonamide) is a uroselective α_1A_-adrenergic receptor (α_1A_-AR) antagonist primarily used for patients with benign prostatic hyperplasia (BPH) presenting with lower urinary tract symptoms [1]. The recommended dose is 0.4 mg or 0.8 mg with a half-life of nine to 15 hours. It is extensively metabolized by the liver.

Currently, α_1_-adrenergic receptor (α_1_-AR) antagonists are commonly prescribed by physicians as first-line agents to treat BPH, a common condition of aging men. α_1_-AR antagonists exert their effects by blocking α_1_-AR-mediated contraction of the prostatic smooth muscle cells and bladder neck [2]. There are three α_1_-AR subtypes: α_1A_, α_1B _and α_1D_. Terazosin, doxazosin and alfuzosin are α_1_-AR antagonists that show equal affinity for all α_1_-AR subtypes [3]. Tamsulosin does not interfere with blood pressure control and has a low potential to cause vasodilation [4]. It is selective for α_1A_-and α_1D_-AR subtypes but has less affinity for the α_1B _subtype.

## Case presentation

A 54-year-old, 80-kg Asian man was scheduled for left thyroid lobectomy for a thyroid nodule. He was clinically euthyroid with normal thyroid function test results. The patient's medical history was insignificant except for BPH. His New York Heart Association classification was class I. For BPH, he was being treated with tamsulosin 0.4 mg at night for one year. He was a smoker and had been smoking approximately 20 cigarettes a day for the past 24 years. He had no known history of allergy to any drug. He consented to general anesthesia for thyroid lobectomy.

Before induction of anesthesia, the patient's recorded blood pressure (BP) was 137/88 mm Hg, heart rate was 72 bpm, and temperature was 36.5°C. Cefazolin 1 g was administered intravenously (IV) in the operating room without any adverse effects. After placement of routine monitors and preoxygenation, general anesthesia was induced with sodium thiopentone 5 mg/kg, pethidine 1 mg/kg and atracurium 0.5 mg/kg, and the patient's trachea was intubated with a reinforced endotracheal tube without any difficulty. The lungs were ventilated with a controlled mode of ventilation with O_2_/N_2_O and 1% isoflurane. The two BP readings taken after induction were 110/70 and 100/64 mm Hg. After laryngoscopy, the patient's maximum BP reading was 162/96 mm Hg, and his heart rate was 104 beats/min. Within 10 minutes, the patient's BP decreased to 75/45 mm Hg despite a rapid infusion of 1 L of lactated Ringer's solution. His heart rate remained at 70 bpm, and his oxygen saturation was between 98% and 99%. End-tidal carbon dioxide remained between 33 and 36 mm Hg, with a respiratory rate of 10 breaths/min. Ephedrine 30 mg in divided IV doses only transiently improved the hypotension (BP to 85/40 mm Hg), and frequent phenylephrine boluses (100 μg/bolus) were required to maintain systolic BP above 90 mm Hg. The patient's skin was warm but not diaphoretic. Skin erythema, urticaria, bronchospasm, facial edema and other features of a potential anaphylaxis or anaphylactoid reaction were absent. No ischemic changes were observed on a three-lead electrocardiogram (ECG). General anesthesia was maintained with O_2_/N_2_O and isoflurane minimum alveolar concentration (MAC) of 0.8%. At this point, the differential diagnosis of hypotension was primarily directed to deep anesthesia or hypovolemia. While these were being addressed, the surgeon was allowed to proceed. There was continuing hypotension requiring phenylephrine. At the time of surgical incision, the patient moved slightly in response to surgical stimulation. The total duration of surgery was 180 minutes (three hours), and the total estimated blood loss was 70 mL. A total of 1200 μg of phenylephrine was required to maintain the systolic a BP above 90 mm Hg throughout surgery.

At the end of surgery, isoflurane was turned off, and the patient's BP increased to 110/70 mm Hg with a heart rate of 75 bpm. The patient completely recovered from neuromuscular blockade after administration of 0.2 mg of glycopyrrolate and 2.5 mg of neostigmine. The patient's trachea was then extubated, and he was shifted to the postanesthesia care unit. He remained in the recovery room for two hours and remained hemodynamically stable with BP in the range of 120/75 to 140/80 mm Hg and heart rate between 70 and 76 bpm.

## Discussion

BPH is common among older men, with approximately 25% of men being affected after 40 years of age [5]. Histologic evidence of the disease is noted in 8% of men in their 30 s, and the prevalence rapidly increases to more than 70% after the age 60 years [6].

All α1-adrenoceptor antagonists have a similar efficacy in improving symptoms and urinary flow. Many of these agents were initially developed and approved for the treatment of patients with hypertension until the development of tamsulosin. Tamsulosin is a more selective α_1A _subtype antagonist, which maintains the α-antagonist effect on the prostatic capsule and bladder neck but has less of an effect on the vascular system and BP. Tamsulosin is not indicated in the treatment of patients with hypertension.

A meta-analysis related to the vascular-related safety profile of α_1 _antagonists was carried out by Nickel et al [7]. They concluded that patients administered alfuzosin, terazosin and doxazosin showed a statistically significant increased risk of developing vascular-related events (dizziness, hypotension or syncope) compared with those taking placebo. Tamsulosin showed only a numerical increase that was not statistically significant.

A MEDLINE search from 1997 to the present revealed no report of any adverse hemodynamic consequences of tamsulosin interaction under anesthesia. Tamsulosin drug interactions have been studied in humans; the use of tamsulosin with nifedipine, enalapril, atenolol, furosemide or digoxin does not require dosage modification when tamsulosin is initiated concomitantly with these agents [8,9]. Tamsulosin is contraindicated with other α1 antagonist and verapamil, which has α_1_-adrenoceptor antagonist effects in therapeutic doses [10]. It is contraindicated in patients with orthostatic hypotension.

This case may illustrate a drug interaction between tamsulosin and inhalational anesthetic agents. Isoflurane is the most likely anesthetic drug to have interacted because the phenylephrine requirement lasted until the end of the case, and the hypotension resolved when isoflurane was stopped. The hypotension in this patient may have been caused by chronic use of tamsulosin. Isoflurane is a known vasodilator and may have synergetic effect with tamsulosin. This may explain why ephedrine was less effective than phenylephrine in counteracting hypotension. Whereas phenylephrine is a pure α_1_-AR agonist, ephedrine has both central and peripheral effects. In the clinical doses administered, its peripheral effects may be less than those of phenylephrine. Ephedrine was administered initially because the differential diagnosis considered at that time was either excessive depth of anesthesia or hypovolemia. When a synergetic effect was suspected, phenylephrine was used instead. The point that negated vasodilation caused by an inappropriate depth of anesthesia was that the patient was receiving less than 1 MAC of isoflurane, and he moved in response to surgical stimulation at the time of incision. Hypovolemia was ruled out when the BP did not rise after administration of 1 L of crystalloid.

The degree of hypotension was exceptional compared with the concentration of anesthetic agent used. The other differential diagnosis for this vasodilated hypotensive state included anaphylaxis and myocardial ischemia. Because the patient did not have any other features of anaphylaxis and had a subsequent negative ECG result for ischemia, these alternative diagnoses were rejected. Other anesthetic drugs used in this patient were thiopentone, pethidine and atracurium. Pethidine and atracurium can both cause histamine release, but no additional clinical signs of histamine release were apparent in this patient. The dramatic increase in BP when isoflurane was turned off goes in favor of some kind of interaction, although the possibility of genetic sensitivity to isoflurane cannot be ruled out completely.

As a result of the recent increase in prescriptions of tamsulosin for aging men with BPH, hypotension under anesthesia associated with this drug may occur. To remove tamsulosin from the circulation would require days of abstinence because tamsulosin has a long half-life of nine to 15 hours.

## Conclusion

This case reports hypotension in a patient on tamsulosin under general anesthesia with N_2_O/O_2_, atracurium and isoflurane anesthesia. Other factors may have contributed to the decreased BP, and tamsulosin cannot be implicated definitively. This case nevertheless demonstrates the importance of vigilance for unexpected hypotension in patients taking tamsulosin. If hypotension does develop in these patients, a direct-acting vasopressor such as phenylephrine should be used.

## Abbreviations

α_1_-AR ANTAGONIST: α_1_-adrenergic receptor antagonist; BPH: benign prostatic hypertrophy; ECG: electrocardiogram; MAC: minimum alveolar concentration; N_2_O: nitrous oxide.

## Consent

Written informed consent was obtained from the patient for publication of this case report. A copy of the written consent is available for review by the Editor-in-Chief of this journal.

## Competing interests

The authors declare that they have no competing interests.

## Authors' contributions

Both authors contributed to the clinical management of the case. The initial draft of the case report was written by DK, and repeated revisions were done by both DK and FAK. Both authors read and approved the final version of the manuscript.
